# Predictors of Diabetes in Older People in Urban China

**DOI:** 10.1371/journal.pone.0050957

**Published:** 2012-11-30

**Authors:** Ruoling Chen, Yiqing Song, Zhi Hu, Eric John Brunner

**Affiliations:** 1 School of Health Administration, Anhui Medical University, Hefei, Anhui, China; 2 Institute of Vascular Medicine, Peking University Third Hospital, Beijing, China; 3 Department of Epidemiology and Public Health, University College London Medical School, London, United Kingdom; 4 Department of Primary Care and Public Health Sciences, King’s College London Medical School, London, United Kingdom; National Institutes of Health - National Institute of Child Health and Human Development, United States of America

## Abstract

**Background:**

China has the largest number of people with diabetes in the world. Over the last 30 years China has experienced rapid economic growth and a growing income gap between rich and poor. The population is ageing, however diabetes in older people has not been well studied to date. In this study we determined incidence and predictors of diabetes in older Chinese people.

**Methods:**

During 2001, using a standard interview method, we examined 1,317 adults aged ≥65 years who did not have diabetes in the city of Hefei, and characterized baseline risk factors. Over 7.5 years of follow up, we documented incident diabetes using self-reported doctor diagnosis and the cause of death in the whole cohort, and HbA_1C_ ≥48 mmol/mol in a nested case-control sample. A multivariate Cox regression model was employed to investigate risk of diabetes in relation to baseline risk factors.

**Results:**

During follow up, 119 persons had newly diagnosed diabetes. World age-standardised incidence of diabetes was 24.5 (95% CI 19.5–29.5) per 1,000 person-years. Risk of diabetes was significantly and positively associated with income, waist circumference and body mass index, smoking and uncontrolled hypertension, but negatively associated with having a hobby of walking and frequency of visiting children/other relatives and contacting neighbours/friends. Higher income was significantly associated with increased diabetes risk regardless of cardiovascular and psychosocial risk factors. Compared to those with middle income and no psychosocial risk factors, the hazard ratio for incident diabetes among participants with high income and psychosocial risk was 2.13 (95% CI 1.02–4.45).

**Conclusions:**

Increasing incidence of diabetes in relation to high income has become an important public health issue in China. Maintaining social networks and gentle physical activities and reducing psychosocial factors may be integrated into current multi-faceted preventive strategies for curbing the epidemic of diabetes in the older population.

## Introduction

In 2011, diabetes affected an estimated 366 million people globally. [1] The number of people with diabetes will continue to increase due to population ageing, population growth, urbanization and increasing prevalence of obesity and sedentary lifestyle. [2] It is estimated to rise to 552 million by 2030, [1] with a 20% increase in adults with diabetes in developed countries and a 69% increase in developing countries. [2] Diabetes is becoming a major cause of morbidity and mortality, leading to a commensurate increase in the social and economic costs. [3].

China is the most populous country in the world, with a population of 1.3 billion. Since its reform in 1978, China has experienced rapid economic growth and an increase in life expectancy, and a population that is ageing. [4] Because of urbanization, change in lifestyle towards westernization such as high calorie diet and sedentary habit, and population ageing, there is concern that a diabetes epidemic has emerged in China. There has been a significant increase in the prevalence of type 2 diabetes during the last three decades, leading to an estimate that China now has the largest number of people with diabetes in the world. [5] A better understanding of the determinants of diabetes is essential if we are to slow this epidemic by identifying preventative measures. Risk factors for diabetes in China have been increasingly explored, mainly in cross-sectional studies undertaken in young and middle aged adult populations. There are few studies which examine predictors of diabetes in older people.

Older Chinese, compared to Caucasians in the West have some unique characteristics and exhibit different patterns of risk factors: clustering extremes of absolute deprivation in earlier age combined with high levels of social support, low levels of depression and low cardiovascular disease risk factors (CVDRFs) except high blood pressure. [6] They have had a rapid increase in income from middle and older ages, and westernisation of lifestyles. Studying such a population may offer insights into the aetiology and prevention of type 2 diabetes. We carried out a population-based cohort study of older people in urban China, [6], [7] having characterized socioeconomic, cardiovascular and psychosocial factors and ascertained diabetes. In this paper, we aimed to determine incidence and predictors of diabetes, specifically examining the combined effects of socioeconomic, psychosocial and cardiovascular factors on diabetes development.

## Methods

### Study Population

In 2001 we selected Hefei city, the capital of Anhui Province, as our study field. Hefei has a population of 2.4 million in an area of 839 km^2^. We chose Yiming sub-district, located in the city centre, with about 50,000 residents within an area of about 0.96 km^2^, to survey as it was our collaborative centre for the community-based health study teams facilitating the data collection. More than 95% of the adult population was employed in government departments, schools, and factories. Average annual personal income was around RMB 12,000 (about US$1,400) at the time. We targeted the population who had lived in the Yiming sub-district ≥5 years and who were aged ≥65 years. We randomly selected 1,810 older people from the resident committee list, of whom 1,736 agreed to participate in the study (response rate 95.9%).

### Interviews at Baseline and Follow Up

The methods of the baseline investigation of the Hefei study have been fully described before. [6] In brief, the participants were interviewed by a trained survey team from the School of Health Administration, Anhui Medical University during 2001 (*wave 1*). Permission for interview and written informed consent was obtained from each participant or, if that was not possible, from the closest responsible adult. Refusals were respected. The main interview materials were a general health and risk factor record and the Geriatric Mental State (GMS). [8] According to a standardized protocol, [9], [10] we measured systolic and diastolic blood pressure, and height, weight and waist circumference.

Using the same protocol, we successfully re-interviewed 1,293 cohort members in 2002 (*wave 2*). [11] In September 2007-March 2009, we re-examined 667 surviving members (*wave 3*) using the GMS questionnaire and health and risk factor record, to which we added more questions including information on dietary intake. In an extended nested case-control study of dementia, [12] we measured HbA_1C_ levels among 16 dementia and 9 non-dementia cohort members. The overall response rate in the follow up from wave 2 and wave 3 was 87.8% after excluding the participants who had moved house or were deceased.

We identified vital status of the cohort members up to December 2008 in this study, including date and causes of death through electronic registration databases from Hefei Center for Disease Control and Police Registration and records from the local resident committees. We used a standard verbal autopsy questionnaire to interview family members, relatives, neighbours or friends of the deceased, which was reviewed by 6 physicians from Anhui Medical University Teaching Hospital to confirm the underlying cause of death. Ethical approval for the cohort study was obtained from the Research Ethics Committee, University College London.

### Risk Factors Recorded at Baseline

The general health and risk factor record contained (1) socio-demographic information, such as educational level, main occupation status, and annual income, (2) social support and relationships, (3) psychosocial aspects, (4) physician-diagnosed cardiovascular diseases and medications and self-assessed physical health, (5) adverse life events occurring in the last two years, and (6) hobbies and activities of daily living (ADL). [13] Regarding annual income, we asked the participant “*Are you satisfied with your income?*”, with a choice of answers at “*Very satisfied*”, “*Satisfied*”, “*Average*” and “*Poor*”. We also asked the participant “*Whether or not you have had serious financial problems in the last two years?*”. [16].

### Ascertainment of Diabetes Outcome

We defined diabetes based on doctor-diagnosis recorded in the general health and risk factor questionnaire at each wave, i.e. those who answered “*Yes*” to the question, “*Have you ever been told by a doctor that you have diabetes?*” In the follow up, we included diabetes identified from the causes of death and those with HbA_1C_ ≥48 mmol/mol in IFCC units (HbA_1C_ ≥6.5% in DCCT units) according to American Diabetes Association Standards [14] with confirmation by a doctor.

### Statistical Analysis

We excluded participants with known diabetes at baseline (n = 175). Distributions of demographic and risk factors between participants with and without incident diabetes were tested by ANOVA for continuous variables and by Chi-square for categorical variables. We computed person-years at risk to the end of follow up, date of death, or date of loss to follow up. The incidence rates of diabetes with 95% confidence intervals (CI) among men and women were calculated and age standardised to the world population 2002–2009 (http://www.census.gov) at ages of 65–69, 70–74, 75–79 and ≥80 years. We used an age- and sex-adjusted Cox regression model to individually assess the associations of baseline risk factors with incident diabetes, calculating hazard ratios (HR) and 95% CIs. A multivariate regression model, which included all variables with p≤0.1 in the age-sex adjusted analysis or potentially important factors (smoking habits), was used to further explore the independent effects of risk factors. To test for trend across income groups, we treated these data as continuous in the model. Due to the rapid economic reform of the 1980s in China, older Chinese may have different patterns of the associations of annual incomes with cardiovascular disease and risk factors and psychosocial factors; for example, richer people may have higher levels of CVDREFs and psychosocial factors, in contrast to the western pattern. Thus we further examined the combined effects of these factors on diabetes. We used a combination variable of income satisfaction and financial problems, [15] i.e. those who had financial problems or poor income were assigned to the lowest income group. Analyses were performed using the SPSS statistical package (Windows version 16.0; SPSS Inc., Chicago, Illinois).

## Results

Of 1,561 participants without diabetes at baseline, 1,317 (mean 72.8, SD 6.1 years; range 65–99) were followed up over a period of 7.5 years (median 3.7 years, 4823.3 person years at risk). One hundred and nineteen people were newly diagnosed with diabetes; 108 cases were documented from doctor diagnosis recorded in the questionnaire, 6 from the causes of death, and 5 from measurement of HbA_1C_ ≥48 mmol/mol. World age-standardized annual incidence of diabetes per 1,000 person years was 24.5 (95% CI, 19.5–29.5), 26.4 (18.7–34.0) in men, 22.6 (16.0–29.2) in women.

Table 1 presents the frequencies of baseline risk factors among participants. Those developing diabetes were more likely to be overweight, have high income (linear trend p = 0.027 across income groups), be married, and suffer from hypochondriasis, uncontrolled hypertension, angina, and hearing problems. Other risk factors included in table 1 and in the general health and risk factors record (data not shown) were not significantly different between participants without and with incident diabetes.

**Table 1:** Distribution of risk factors for incident diabetes in older people – Hefei cohort study, China.

Variable	DiabetesN = 119		Non-DiabetesN = 1198		
	n	(%)	n	(%)	P
***Basic characteristics***					
**Age (years)**					
65–69	36	30.3	465	38.8	0.177
70–74	47	39.5	365	30.5	
75–79	21	17.6	219	18.3	
≥80	15	12.6	149	12.4	
**Sex**					
Women	54	45.4	632	52.8	0.124
Men	65	54.6	566	47.2	
**Weight (kg)**					
Mean (SD)	65.9	11.1	61.7	11.3	<0.001
**Waist circumference (cm)**					
Mean (SD)	92.4	12.2	87.8	12.4	<0.001
**BMI (kg/m^2^)**(Chinese definition)					
<18.5	4	3.4	83	6.9	0.025
18.5–23.9	45	37.8	579	48.3	
24.0–27.9	54	45.4	421	35.1	
≥28	16	13.4	115	9.6	
**Smoking habits**					
Never-smoking	53	44.5	631	52.7	0.079
Ex-smoking	11	9.2	64	5.3	
Current-smoking	28	23.5	236	19.7	
***Socio-economic position and hobby***					
**Annual income**					
Poor/final problems	2	1.7	199	16.6	0.143
Average	14	11.8	767	64.7	
Satisfactory	77	64.7	162	11.8	
Very satisfactory	26	21.8	79	1.7	
**Educational level**					
≥High secondary school	52	43.7	522	43.6	0.990
Secondary school	27	22.7	283	23.6	
Primary school	18	15.1	184	15.4	
Illiterate	22	18.5	209	17.4	
**Main occupation**					
Officer/teacher	33	27.7	694	57.9	0.986
Business/other (housewife)	68	57.1	178	14.9	
Manual labourer/Peasant	18	15.1	326	27.2	
**Walking**					
No	35	29.4	265	22.1	0.070
Yes	84	70.6	933	77.9	
***Social network***					
**Marital status**					
Married	101	84.9	897	74.9	0.041
Never married	1	0.8	7	0.6	
Widowed	17	14.3	294	24.5	
**Living with**					
Spouse and/or grand/children and/or parents	34	28.6	346	28.9	0.087
Spouse only	62	52.1	507	42.3	
Children and/or Grant children only	14	11.8	166	13.9	
Others	6	5.0	74	6.2	
No-one	3	2.5	105	8.8	
**Frequency of visiting children or other relatives**					
Daily	40	33.6	510	42.6	0.164
At least weekly -<monthly	57	47.9	457	38.1	
Monthly	16	13.4	181	15.1	
Never	6	5.0	50	4.2	
***Psychosocial factors***					
**Worrying**					
No	97	81.5	992	83.0	0.679
Yes	22	18.5	203	17.0	
**Hypochondriasis**					
No	100	84.0	1089	91.0	0.014
Yes	19	16.0	108	9.0	
**Anxiety/depressive syndrome level (GMS-AGECAT)**					
0	95	79.8	1022	85.3	0.113
1–5	24	20.2	176	14.7	
***Adverse Life Events occurring within two years***					
**Death of closely related people**					
No	110	92.4	1129	94.2	0.427
Yes	9	7.6	69	5.8	
***Cardiovascular disease and risk factors***					
**Hypertension (BP≥160/95 mmHg or taking antihypertensive drugs)**					
***Non-hypertension***(<140/90 mmHg)	45	37.8	503	42.0	0.035
(≥140/90 and <160/95 mmHg)	21	17.6	234	19.5	
***Hypertension***					
Undetected	4	3.4	79	6.6	
Untreated	2	1.7	35	2.9	
Uncontrolled	20	16.8	99	8.3	
Controlled	27	22.7	248	20.7	
**Hypercholesterolemia**					
No	97	82.2	1041	88.4	0.048
Yes	21	17.8	136	11.6	
**Angina**					
No	109	92.4	1149	96.5	0.028
Yes	9	7.6	42	3.5	
***Other health status***					
**Hearing problems**					
No	88	74.6	998	83.6	0.013
Yes	30	25.4	196	16.4	
**Activity of daily living (score)** §					
0	101	84.9	1056	88.1	0.116
1–3	12	10.1	66	5.5	
≥4	6	5.0	76	6.3	

§ the participant reported their level of difficulty in questions of ADL scale. The valid response was ‘no difficulty alone’ (score 0), ‘manages alone with difficulty (score 1) cannot do alone (score 2). The scale consists of 14 items: having a bath or all-over wash, washing hands and face, putting on shoes and stockings/socks, doing up buttons and zips, dressing yourself other than the above, getting to and using the WC, getting in and out of bed, feeding self, shaving (men) or doing hair (women), cutting your own toenails, getting up and down steps, getting around the house, going out of doors alone and taking medicine.

In the age- and sex-adjusted analysis (Table 2), older age, body weight, body mass index (BMI), waist circumference, uncontrolled hypertension and other traditional CVDRFs were significantly associated with diabetes risk. For socioeconomic and psychosocial factors, diabetes risk was associated with higher income (trend p = 0.030 across 4 groups), being married, living in a big family, having hypochondriasis, and having anxiety/depressive syndromes (borderline significance). The risk reduced with daily visiting children/relatives (and also daily contacting neighbours and friends in the communities – data not shown) and having a hobby of walking, and increased with hearing problems and low/middle ADL score (Table 2).

**Table 2:** Age-and sex-adjusted hazard ratios for incident diabetes in older people – Hefei cohort study, China.

Variable	HR	95% CI	P
***Basic characteristics***			
**Age (years)**			
65–69	1.00		
70–74	1.60	(1.04–2.48)	0.034
75–79	1.27	(0.74–2.18)	0.378
≥80	1.65	(0.90–3.03)	0.104
**Sex**			
Women	1.00		
Men	1.28	(0.89–1.84)	0.183
**Weight (kg)**			
Mean (SD)	1.03	(1.02–1.05)	<0.001
**Waist circumference (cm)**			
Mean (SD)	1.03	(1.01–1.04)	<0.001
**BMI (kg/m^2^)** (Chinese definition)			
<18.5	0.76	(0.27–2.13)	0.604
18.5–23.9	1.00		
24.0–27.9	1.89	(1.26–2.82)	0.002
≥28	1.92	(1.08–3.40)	0.026
**Smoking habits**			
Never-smoking	1.00		
Ex-smoking	0.60	(0.29–1.25)	0.173
Current-smoking	1.08	(0.53–2.19)	0.832
***Socio-economic position and hobby***			
**Annual income**			
Poor/financial problems	1.00		
Average	3.12	(0.69–14.1)	0.140
Satisfactory	3.49	(0.84–14.5)	0.086
Very satisfactory	4.66	(1.08–20.2)	0.040
**Educational level**			
≥High secondary school	1.00		
Secondary school	1.03	(0.64–1.64)	0.918
Primary school	1.03	(0.59–1.80)	0.928
Illiterate	1.29	(0.75–2.23)	0.361
**Main occupation**			
Officer/teacher	1.00		
Business/other (housewife)	1.17	(0.68–2.01)	0.565
Manual labourer/Peasant	1.10	(0.71–1.70)	0.671
**Walking**			
No	1.00		
Yes	0.66	(0.44–0.99)	0.047
***Social network***			
**Marital status**			
Married	1.00		
Never married	0.85	(0.12–6.16)	0.873
Widowed	0.53	(0.30–0.93)	0.026
**Living with**			
Spouse and/or grand/children and/or parents	1.00		
Spouse only	1.18	(0.77–1.79)	0.453
Children and/or Grant children only	0.84	0.431.65	0.615
Others	0.94	(0.39–2.30)	0.895
No-one	0.30	(0.09–0.97)	0.045
**Frequency of visiting children or other relatives**	0.30	(0.09–0.97)	0.045
Daily	0.66	(0.44–1.00)	0.049
At least weekly -<monthly	1.00		
Monthly	0.66	(0.38–1.16)	0.148
Never	0.74	(0.32–1.71)	0.476
***Psychosocial factors***			
**Worrying**			
No	1.00		
Yes	1.15	(0.72–1.83)	0.567
**Hypochondriasis**			
No	1.00		
Yes	1.79	(1.09–2.94)	0.021
**Anxiety/depressive syndrome level (GMS-AGECAT)**			
0	1.00		
1–5	1.56	(0.99–2.45)	0.055
***Adverse Life Events occurring within two years***			
**Death of closely related people**	1.00		
No	1.00		
Yes	1.20	(0.61–2.38)	0.593
***Cardiovascular disease and risk factors***			
**Hypertension (BP≥160/95 mmHg or taking antihypertensive drugs)**			
***Non-hypertension***(<140/90 mmHg)	1.00		
(≥140/90 and <160/95 mmHg)	0.94	(0.56–1.59)	0.825
***Hypertension***			
Undetected	0.68	(0.24–1.89)	0.457
Untreated	0.61	(0.15–2.54)	0.501
Uncontrolled	1.90	(1.12–3.22)	0.017
Controlled	1.16	(0.72–1.88)	0.534
**Hypercholesterolemia**			
No	1.00		
Yes	1.58	(0.98–2.55)	0.058
**Angina**			
No	1.00		
Yes	1.74	(0.88–3.45)	0.113
***Other health status***			
**Hearing problems**			
No	1.00		
Yes	1.63	(1.07–2.49)	0.024
**Activity of daily living (score)**			
0	1.00		
1–3	1.97	(1.07–3.63)	0.029
≥4	1.05	(0.45–2.44)	0.912

In the multivariate analysis (Table 3), we still observed that the risk of diabetes significantly increased with BMI, and with waist circumference (HR 1.02, p = 0.025), income (trend p = 0.042), smoking and uncontrolled hypertension, but reduced with walking and visiting children/other relatives daily.

**Table 3:** Multivariate analysis for predictors of incident diabetes in older people - Hefei cohort study, China.

Baseline variable	Multivariate analysis		
	HR†	95%CI	p
***Basic characteristics***			
**Age (years)**			
65–69	1.00		
70–74	1.34	(0.85–2.12)	0.210
75–79	1.13	(0.65–1.97)	0.676
≥80	1.71	(0.86–3.40)	0.129
**Body mass index (BMI, kg/m^2^)**			
<18.5	0.63	(0.22–1.82)	0.393
18.5–23.9	1.00		
24.0–27.9	1.81	(1.19–2.76)	0.005
≥28	1.77	(0.97–3.25)	0.065
***Socio-economic position, lifestyles, and hobby***			
***Annual income***			
Average/poor/financial problem	1.00		
Satisfactory	1.16	(0.65–2.06)	0.617
Very satisfactory	1.69	(0.85–3.33)	0.134
**Smoking habits**			
Never-smoking	1.00		
Ex-smoking	1.31	(0.68–2.54)	0.424
Current-smoking	1.75	(1.08–2.81)	0.022
**Walking**			
No	1.00		
Yes	0.55	(0.35–0.86)	0.009
***Social network***			
**Marital status**			
Married	1.00		
Widowed (including several divorcees)	1.22	(0.14–10.74)	0.856
Never married	0.58	(0.24–1.43)	0.239
**Living with**			
Spouse and/or grand/children and/or parents	1.00		
Spouse only	1.04	(0.65–1.65)	0.881
Children and/or Grant children only	1.56	(0.59–4.15)	0.370
Others	1.08	(0.38–3.09)	0.881
No-one	0.32	(0.09–1.19)	0.089
**Frequency of visiting children or other relatives**			
Daily	0.56	(0.35–0.90)	0.016
At least weekly -<monthly	1.00		
Monthly	0.92	(0.37–2.32)	0.867
Never	0.75	(0.42–1.33)	0.322
***Psychosocial factors***			
**GMS-AGECAT syndrome level**			
Neither depressive nor anxiety syndromes	1.00		
Any depressive or anxiety syndromes	1.36	(0.82–2.26)	0.239
**Hypochondriasis**			
No	1.00		
Yes	1.41	(0.78–2.55)	0.256
***Cardiovascular disease and risk factors***			
**Hypertension (BP≥160/95 mmHg or taking antihypertensive drugs)**			
***Non-hypertension***(<140/90 mmHg)	1.00		
(≥140/90 and <160/95 mmHg)	0.88	(0.51–1.51)	0.635
***Hypertension***			
Undetected	0.63	(0.22–1.79)	0.382
Untreated	0.55	(0.13–2.30)	0.413
Uncontrolled	1.80	(1.03–3.14)	0.040
Controlled	0.83	(0.50–1.39)	0.482
**Hypercholesterolemia**			
No	1.00		
Yes	1.33	(0.80–2.21)	0.273
***Other health status***			
**Hearing problems**			
No	1.00		
Yes	1.54	(0.97–2.44)	0.066
**Activity of daily living (score)**			
0	1.00		
1–3	1.63	(0.84–3.18)	0.151
≥4	0.55	(0.21–1.47)	0.231

† adjusted for smoking and all variables which had significant level < = 0.100 from age-sex adjustment analysis in table 2 (BMI or Waist circumference).

Table 4 shows risk of incident diabetes in subgroups stratified by income group, and cardiovascular and psychosocial factor status. When income was combined with CVDRFs, participants with the highest income regardless of CVDRFs had an increased risk of diabetes and, in those with middle and low incomes, the CVDRFs increased the risk of diabetes. As compared with participants who had middle income and no psychosocial risk factors, only these having the highest income and psychosocial risk had a significantly increased risk of diabetes (Table 4).

**Table 4:** Hazard ratios (HR) for incident diabetes by income group and cardiovascular or psychosocial risk factors in older people –Hefei cohort study, China.

				Income group					
	High@1			Middle@2			Low@3		
	Nos (%)*	HR† (95%CI)	P	Nos (%)*	HR† (95%CI)	p	Nos (%)*	HR† (95%CI)	p
**Cardiovascular risk factors** §									
No	3/27(11.1)	4.60(1.20–17.7)	0.026	8/196(4.1)	1.00		1/53(1.9)	0.60(0.07–4.82)	0.629
Yes	23/198(11.6)	2.75(1.19–6.35)	0.018	69/648(10.6)	2.45(1.18–5.12)	0.017	15/195(7.7)	1.92(0.80–4.65)	0.147
**Psychosocial factors$**									
No	7/70(10.0)	2.19(0.86–5.61)	0.102	13/228(5.7)	1.00		2/38(5.3)	0.95(0.21–4.32)	0.945
Yes	19/155(12.3)	2.13(1.02–4.45)	0.045	64/616(10.4)	1.77(0.96–3.26)	0.066	14/210 (6.7)	1.38(0.64–2.99)	0.416

@1High income- Very satisfactory income;

@2Middle income - Satisfactory income;

@3low - Average/Poor income or Financial problem in the last two years.

* Nos of Diabetes/participants (%).

§ includes BMI>24, current- and ex- smoking, hypertension, hypercholesterolemia and no hobby of walking.

$ including any of worrying, feeling lonely, hypochondriasis, anxiety/depressive syndrome level, death of closely related people, horrifying experience, and less than daily visiting children/other relatives.

† *In the combined analysis of income with cardiovascular risk factors*, adjusted for age, marital status, living status, frequency of visiting children/other relatives, hypochondriasis, anxiety/depressive syndrome levels, hearing problems, and activity of daily living. *In the combined analysis of income with psychosocial factors*, adjusted for age, BMI, smoke habits, hobby of walking, hypertension, hypercholesterolemia, hearing problems and activity of daily living.

The combined analysis of cardiovascular and psychosocial factors showed no significant differences in the risk of diabetes among the four groups (Table S1) but, compared with all participants having no CVDRFs, the HRs in participants having CVDRFs without and with psychosocial factors was 1.44 (0.70–2.99) and 2.36 (1.29–4.34) respectively.

## Discussion

In a population of older people in China, we observed a higher incidence of diabetes in comparison with Caucasian counterparts in the West. [16]–[17] Apart from the traditional risk factors, the risk of diabetes was positively associated with higher income and psychosocial factors, and inversely associated with the hobby of walking and daily visiting children/relatives and neighbours/friends in the community. There was a more than doubled risk of developing diabetes in participants who had the highest income and CVDRFs or psychosocial factors and in those who had both CVDRFs and psychosocial factors.

Among older Caucasian populations in the West, incidence of diabetes is about 10 per 1,000 person years. [17]–[18] [16], [19]–[20] Compared to this, the incidence of diabetes in the present study is higher. High diabetes incidence in older Chinese has also been observed in a study undertaken in north of China. [21] Together with rapid economic development and population ageing [4] our finding suggests an emerging epidemic and rising burden of diabetes in China, making it an important public health problem. Our study indicates the potential importance of further actions to prevent diabetes in the older adults community. In the current study, a number of factors predicted the development of diabetes. Many of these, including obesity, smoking and hypertension, [22] are well known. However, the association of income with incident diabetes is less investigated and not well understood. Recently, Guize et al [23] observed that socioeconomic deprivation increased the risk of diabetes in a French population. In contrast, our study showed that higher income was consistently associated with higher diabetes incidence. The conflicting finding to that in the West could be due to special characteristics of our study population. Of note, the participants in this study suffered long-term absolute poverty from birth to the end of 1970s, having experienced substantial social, cultural and economic revolution including a 3-year period of starvation (1960–62) [24]. In utero and early life these conditions may have affected growth and organ development with later adverse consequences for glucose homeostasis. [25] Since the economic reform of 1978, annual incomes have increased and lifestyle and dietary habits have changed with increased consumption of cigarettes, alcohol and meat, and increased sedentary activity. The changes may contribute to unhealthy western dietary patterns and over-consumption of foods or total calorie intake. Further, we observe that those who became rich in China are not necessarily highly educated, and may have changed their lifestyles adversely without awareness of the health risks. Our baseline data show high correlations between higher incomes and higher prevalence of CVDRFs (e.g. prevalence of BMI≥26 kg/m^2^ in four income groups from very satisfactory to poor income was 29%, 24%, 21%, and 16%, trend p<0.001), a pattern opposite to that generally observed in the West. [9] Furthermore, the *wave 3* dietary intake data showed a significant association of baseline income with egg, meat and fish consumption. Prevalence of eating eggs ≥ once per day was 41.2%, 50.6%, 78.0% and 79.3% among participants who had baseline poor income (n = 34), average income (n = 174), satisfactory income (n = 318) and very satisfactory income (n = 92). The corresponding figures for meat consumption were 17.6%, 12.6%, 31.8%, and 37.0%, and for fish consumption 8.8%, 8.6%, 19.8%, and 17.4% respectively. It is possible that these correlations could partly explain the association between higher income and increased diabetes risk.

To our knowledge, this study is the first to examine psychosocial factors as predictors of diabetes development in an older population and to report the findings of the lower diabetes risk in people frequently visiting children/other relatives and contacting friends in the communities and neighbours. High levels of social engagement may indicate relatively high levels of vitality and may in addition reduce the risk of depression, which has been related to diabetes risk. [26] In western countries where depressive disorders are common, [13] many but not all studies have shown that a history of depression was a risk factor for type 2 diabetes. [26] Some investigators [27] questioned the aetiological role of depression in developing diabetes. In Sweden, Eriksson et al [28] found that psychological distress including symptoms of anxiety and depression increased the risk of diabetes in middle-aged men but not in women. The Whitehall II study suggested that psychosocial work stress increased the risk of diabetes in middle-aged women but not in men. [29] In spite of these controversies our study identified a significant relationship between incident diabetes and baseline psychosocial factors (components including worrying, anxiety/depressive symptoms and less frequently visiting children/relatives) among those having higher income.

The finding of a lower diabetes risk in persons frequently having contact with children, other relatives, friends and neighbours, which could reflect aspects of Chinese culture and social networks, is significant. The large multigenerational household containing older family members is a part of Chinese culture and tradition. Traditional sayings in China include: "Bringing up a son will prevent ageing," and “One should not go far away from your parents”. Urban Chinese tend to live closely in high density housing and apartments, and within the community there are often active senior residents’ clubs for elders to play poker and other games and to talk. With this culture and tradition, some study participants may live with their children, have relatives living nearby, and frequent contact with neighbours and friends. This may reflect good family and social relationships, leading to a low risk of depression. [30] Daily contact with children, other relatives, neighbours and friends may also encourage physical activity, thereby reducing risk of diabetes.

Our study further showed that participants who liked walking had a lower risk of developing diabetes than those without such a hobby. The habit of walking reflects the Chinese tradition, embodied in the old saying: “Walking for 99 steps after meal one can live until 99 years old”. The study finding suggests that maintaining gentle physical activities and improving traditional social network and contacts in late life may be integrated into current multi-faceted preventive strategies for curbing the epidemic of diabetes.

This study has several strengths. First, our data provided evidence regarding the aetiological role of socioeconomic, cardiovascular and psychosocial factors in development of diabetes in older people in the world’s most populous developing country which is experiencing a rapid transition from poverty to economic development. Recent studies in the West show that diabetes is related to low socioeconomic status (SES) and cardiovascular risk factors. [31] Psychosocial factors including depression and stress may also be important. [26], [27], [29] Few studies have specifically investigated the combined effects of income, psychosocial and cardiovascular factors on diabetes development. We examined the combined effects of these risk factors. Second, we had relatively high response rates to standardized face-to-face interviews at both baseline and follow up. The study has some limitations. The sample size in the study was not large, which yielded wider confidence intervals for some risk factors in relation to diabetes risk. We identified a number of significant risk factors, however our findings were more conservative when multivariate modelling was applied. We have not directly validated the doctor-diagnosed diabetes diagnosis reported by participants. Previous studies [16] suggest that self-reported diabetes in older people is reliable. Hefei city has several university teaching hospitals, population-based health education campaigns are common and community health care systems have been established. Older residents are encouraged to have health examinations regularly, which including measurement of blood glucose. Thus, under-diagnosis of diabetes in the cohort is likely to be minimal. Lastly, while the study region had comparable levels of economic development and modernization to other urban provinces, caution should be exercised in generalising our findings to China’s 130 million older inhabitants, particularly in rural areas.

In summary, there was an increased incidence of diabetes in older people whose early life was marked by extreme poverty, but in mid- and older ages experienced rapid economic improvements. In addition to traditional risk factors and some health-related problems, the risk for developing diabetes was significantly increased with high income and psychosocial factors. CVDRFs in combination with psychosocial factors increased the risk of developing diabetes. The association of higher income with incident diabetes may be in part be due to high intake of meats and calories. A composite psychosocial factor was related to increased diabetes risk among people who had highest incomes or CVDRFs. Further exploration of Chinese culture and tradition and rapid economic growth associated with lifestyle changes may yield new insights into preventive strategies for type 2 diabetes.

## Supporting Information

Table S1 **Number of incident diabetes and Hazard ratio (HR) for combined cardiovascular risk factors and psychosocial factors in older people – Hefei cohort study, China.**
(DOCX)
