# Supplementary material for: Predictors of Diabetes in Older People in Urban China
Source: PLoS One. 2012 Nov 30;7(11):e50957. doi: 10.1371/journal.pone.0050957 (PMC3511385; doi:10.1371/journal.pone.0050957)
Supplement: Table S1 — Number of incident diabetes and Hazard ratio (HR) for combined cardiovascular risk factors and psychosocial factors in older people – Hefei cohort study, China. (DOCX) [file pone.0050957.s001.docx]

**Supplementary Table 1 - Number of incident diabetes and Hazard ratio (HR) for combined cardiovascular risk factors and psychosocial factors in older people – the Hefei cohort study, China.**

|  | **Psychosocial factors** | | | | | | |
| --- | --- | --- | --- | --- | --- | --- | --- |
|  | No | | |  | Yes | | |
| **Cardiovascular risk factors** | Nos of Diabetes /participants (%) | HR (95%CI)† | p |  | Nos of Diabetes /participants (%) | HR (95%CI)† | p |
|  |  |  |  |  |  |  |  |
| No | 3/71 (4.2) | 1.00 |  |  | 9/205 (4.4) | 0.98 (0.26-3.65) | 0.976 |
|  |  |  |  |  |  |  |  |
| Yes | 19/265 (7.2) | 1.42 (0.46-4.84) | 0.572 |  | 88/776 (11.3) | 2.32 (0.73-7.44) | 0.154 |
|  |  |  |  |  |  |  |  |

†adjusted for age, income, and activity of daily living.
